# The role of the ventral striatum in the relationship between impulsive decision-making and emotional self-regulation by cognitive reappraisal

**DOI:** 10.1038/s41598-025-13599-8

**Published:** 2025-07-30

**Authors:** Youngwoo Bryan Yoon, Wi Hoon Jung

**Affiliations:** 1 Department of Psychiatry, Columbia University Irving Medical Center, New York, NY USA; 2 New York State Psychiatric Institute, New York, NY USA; 3 Department of Psychology, Gachon University, 1342 Seongnam-daero, Sujeong-gu, Seongnam-si, Gyeonggi-do 13120 South Korea

**Keywords:** Delay discounting, Emotion regulation, Intertemporal choice task, Valuation, Reward system, Ventral striatum, Magnetic resonance imaging, Decision, Human behaviour, Nervous system, Behavioural methods, Medical research, Neurology, Risk factors

## Abstract

Delay discounting (DD), the preference for smaller, immediate rewards over larger, delayed ones, is a key measure of temporal impulsivity. While its link to behavioral self-regulation is well-studied, the relationship with emotional self-regulation is less understood. This study explored this relationship and its neuroanatomical mediators in the brain’s reward system. We administered the Emotion Regulation Questionnaire (ERQ) and a DD task to 155 young adult college students and collected structural MRI data. Our data revealed that greater use of cognitive reappraisal as an emotion regulation strategy was significantly associated with lower DD rates (i.e., reduced temporal impulsivity). No such relationship was found for expressive suppression. Furthermore, mediation analysis showed that higher cognitive reappraisal scores were associated with lower gray matter volume in the left ventral striatum, which in turn predicted lower DD. While the pattern of results is statistically consistent with full mediation, the cross-sectional nature of our data precludes causal inference. In conclusion, these results identify a novel neuroanatomical mechanism for temporal impulsivity. They suggest that cognitive reappraisal helps control impulsive choice and the process is mediated by the ventral striatum. This may provide a useful biomarker for developing interventions to improve self-control.

## Introduction

Humans make decisions based on the subjective value they place on the available rewards^1^. Delay discounting (DD, quantified as discount rate, *k*) is a measure of individual differences in the preference for smaller, immediate rewards versus larger, delayed ones^2^. Understanding the mechanisms underlying DD allows researchers to pinpoint the cognitive, behavioral, and neurobiological factors that drive impulsive decision-making^3^. This knowledge is particularly valuable in addressing poor behavioral control, in which impulsive decisions often result in adverse long-term outcomes^4^. Insights from DD research can guide the development of interventions aimed at increasing self-control and improving decision-making strategies. It can also contribute to the creation of effective treatments for psychiatric conditions such as substance use disorders, attention deficit hyperactivity disorder, and personality disorders^5–9^.

Self-regulation is central to DD^10^. Research indicates that those with stronger self-regulatory abilities are more likely to delay gratification, as they have greater impulse control and choose greater, long-term benefits over smaller, immediate rewards^11^. These findings suggest that enhancing emotional and behavioral self-regulation could improve outcomes in fields where DD is key, such as addiction recovery, financial planning, and health^12^. Research has tended to emphasize the role of behavioral self-regulation in the control of impulsive choices^13^, in which the dorsolateral prefrontal cortex (DLPFC) plays a critical role^14^. Although less studied, emotional self-regulation skills are also important to the reduction of impulsivity and improved decision-making. Emotional self-regulation can involve managing the stress and negative emotions resulting from delayed gratification and enhancing the emotional value of delayed rewards using the imagination^15^. However, the relationship between emotional self-regulation and DD remains underexplored.

Cognitive reappraisal and expressive suppression are key emotional self-regulation strategies^16^. The Emotion Regulation Questionnaire (ERQ) is commonly used to assess these strategies, with higher scores on particular dimensions indicating more frequent use of specific strategies^17^. Individuals with effective emotional self-regulation skills are better at managing emotional impulses^18^. Conversely, poor emotional self-regulation leads to a higher likelihood of choosing immediate gratification due to difficulties coping with the discomfort or anxiety of waiting. Although previous research has established a link between greater DD and overall emotion dysregulation^19^, the specific relationships between DD and emotional self-regulation strategies, such as cognitive reappraisal and expressive suppression, remain unexplored.

Emotional responses to external stimuli are processed by a network of brain regions, with the limbic system, particularly the amygdala, playing a crucial role in the initial detection and generation of emotional responses^20,21^. Effective emotional self-regulation often involves the prefrontal cortex modulating activity within these limbic structures^22,23^. Beyond these core emotional circuits, areas within the brain’s reward system are also critically involved. This system, including the ventral striatum (VS) and medial prefrontal cortex (MPFC), contributes to processing incentive motivation and positive expectations^24–27^, and plays an important role in integrating emotional information to determine the value of stimuli and guide decision-making^28,29^. Given the established role of the reward system in DD, we hypothesized that focusing on these areas could reveal underlying neural markers of emotional self-regulation, which is closely linked with DD^30^. Therefore, we predicted that the VS and the MPFC are involved in both the reward valuations related to DD and emotional self-regulation.

While many investigations into these processes have utilized functional neuroimaging, there is also evidence suggesting that individual differences in brain structure, such as gray matter volume (GMV) in these reward- and regulation-related regions, are linked to dispositional traits like impulsivity and the capacity for self-regulation^31–36^. Such structural variations may reflect stable individual differences in the underlying neural architecture supporting these functions^31,37^. Therefore, in this study, we used voxel-based morphometry (VBM) to investigate potential neuroanatomical mediators.

**Fig. 1:**
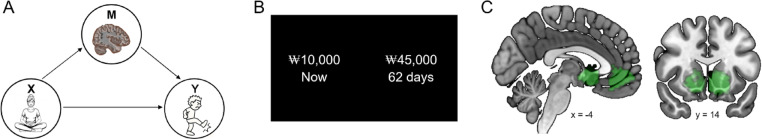
Intertemporal choice task and the associated areas of the brain’s reward system. (**A**) Schematic diagram illustrating the hypothesis. X represents the independent variable (emotion regulation), Y the dependent variable (impulsive choice), and M the mediating variable (gray matter volume); (**B**) Example task trial. Participants were asked to make a series of 120 choices between a smaller immediate reward (e.g., 10,000 won now) and a larger delayed reward (e.g., 45,000 won in 62 days); (**C**) The areas belonging to the brain’s reward system and delay discounting, shown in green. These include the ventral striatum and the medial prefrontal cortex, which are both components of the valuation network. The green areas were identified in a previous meta-analysis that examined the neural correlates of the subjective valuation of rewards^30^. We used these areas to create a binary mask for small-volume multiple comparison corrections.

To explore the neuroanatomical mediators of the relationship between emotional self-regulation and DD, we applied VBM to structural magnetic resonance imaging (MRI) data. We hypothesize that an individual’s ability to regulate their emotions—specifically greater use of cognitive reappraisal (as measured by ERQ subscale scores)—will be associated with significantly *lower* DD (log-transformed *k* score, as a dependent variable). In addition, we propose that, as neuroanatomical structures involved in the brain’s reward system and goal-directed behavior, the GMV of the VS and/or MPFC (as a mediator) will mediate this relationship (Fig. 1A).

## Methods

### Participants

A total of 160 young adult participants were recruited for this study on the neural mechanisms of impulsivity and risk-taking. All participants were young adult college students who were excluded if they had a known history of a psychotic disorder, neurological illness, substance abuse, or significant head injury. Participants completed behavioral tasks, including an intertemporal choice task (used as a measure of DD)^38^, a risk tolerance task^39^, and several psychological scales. All tasks, including imaging, were completed on the same day. All participants also underwent brain scans. These consisted of a high-resolution T1-weighted anatomical MRI, a resting-state functional MRI (fMRI), and diffusion tensor imaging. Two of the participants were excluded from the sample due to missing data (T1-weighted MRI, ERQ scores, and DD). A further three were excluded based on the homogeneity of the 3D gray matter images obtained after VBM preprocessing. Therefore, 155 participants were included in our final analysis. Participants had a mean (± SD) age of 22.56 (± 2.76). They consisted of 78 females and 77 males. There were 150 right-handed and five left-handed participants. The mean (± SD) duration of education was 15.11 (± 1.39) years. All participants provided written informed consent to participation. The study was conducted in accordance with the tenets of the 2013 revision of the Declaration of Helsinki and all study procedures were approved by the Institutional Review Board of Gachon University.

### Task

The DD task (also known as the intertemporal choice task) presents participants with a series of 120 choices on a screen. This fixed set of 120 trials was identical for all participants to allow for direct comparison of individual *k*-values derived from their choices. For each, they are asked to choose between a smaller immediate financial reward, fixed at ₩10,000 for all trials, and a variable larger, delayed financial reward (Fig. 1B). The monetary amounts were presented in the local currency, KRW (₩10,000 is around 8–9 USD). The amount of the larger, delayed reward ranged from ₩11,000 to ₩48,000. The delay ranged from 2 to 180 days. The discounting rate (*k*) was estimated using a hyperbolic model^38,40^. We assumed that each individual’s decisions were a stochastic function of the difference in the subjective value (*SV*) they placed on the two options. The following logistic function was used to transform the *SV* for each of the two options on each trial into a probability of choosing a given option, as follows:

$$\:{P}_{1}=\:\frac{1}{1+{e}^{-\beta\:\left(SV1-SV2\right)}},\:{P}_{2}=1-\:{P}_{1}$$

where *P*_1_ and *P*_2_ refer to the probability that participants chose the delayed option and the immediate option, respectively. *SV1* and *SV2* refer to the participant’s estimated SV of the delayed and immediate options, respectively. *β* was used as a scaling factor and was fitted for each participant.

$$SV = A \cdot \left( {1{\text{ }} + kD} \right)^{{ - 1}}$$

Based on the hyperbolic model, we assume that *SV* is a hyperbolic function of the reward amount (*A*) and delay (*D*). *k* is the participant’s DD rate. Larger *k* values denote a greater degree of discounting future rewards. Before performing our statistical analyses, the *k* values were log-transformed to normalize the distribution.

### Emotional regulation questionnaire

The ERQ, developed by Gross and John (2003), is composed of ten items measuring two subscales: cognitive reappraisal and expressive suppression^17^. It is a widely used questionnaire for assessing these strategies and has been validated in various populations, including young adults^41–43^. The items are presented as statements that participants rate according to the degree to which each statement describes them. The cognitive reappraisal subscale includes six items (e.g., “I control my emotions by changing the way I think about the situation I’m in.”) and expressive suppression includes the other four items (e.g., “I control my emotions by not expressing them”)^17^. It captures individuals’ (self-perceived) tendencies to use these two regulation strategies. Items are answered on 7-point Likert scales ranging from 1 (strongly disagree) to 7 (strongly agree). The higher the score, the greater the use of that emotional self-regulation strategy.

### Data acquisition and preprocessing

Imaging data were obtained using a 3-T Trio MRI scanner (Siemens, Erlangen, Germany). High-resolution T1-weighted anatomical images were acquired using a 3D magnetization-prepared rapid gradient echo sequence (repetition time = 1900 ms, echo time = 2.52 ms, flip angle = 9°, voxel size = 1.0 × 1.0 × 1.0 mm^3^ 192 sagittal slices). Using high-resolution T1-weighted anatomical images, we performed a VBM analysis to estimate GMV across the brain. These GMV estimates served as the key neuroanatomical variable in our subsequent regression and mediation analyses, which investigated the structural brain correlates of cognitive reappraisal and its relationship with DD.

Imaging data preprocessing and all statistical analyses were conducted using MATLAB R2023a (The MathWorks, Inc., Natick, MA, USA). Data for VBM analysis were preprocessed using the Computational Anatomy Toolbox (CAT) (http://www.neuro.uni-jena.de/cat) for SPM12 (http://www.fil.ion.ucl.ac.uk/spm/) set to its default options. In brief, all 3D T1-weighted anatomical images were corrected for bias-field inhomogeneity and were segmented into gray matter, white matter, and cerebrospinal fluid images. Next, the geodesic shooting algorithm was applied to normalize and modulate the gray matter images^44^. Total intracranial volume (TIV) was estimated as the sum of the total GM, WM, and CSF volumes. After visual inspection of the data quality, the images were smoothed with 6-mm full width at half maximum Gaussian kernel. The sample homogeneity of the 3D VBM data was assessed using the data quality function for VBM data homogeneity, implemented in the CAT.

### Statistical analysis

To determine whether there is a relationship between ERQ scores and DD, we performed Spearman correlation analyses between scores on each ERQ subscale and DD, controlling for age, sex, handedness, and duration of education. The significance level was set at *p* < 0.05.

As expressive suppression was not found to correlate with DD, we aimed to determine the neuroanatomical correlates of cognitive reappraisal scores only. This was achieved by applying multiple regression analyses, implemented in SPM12, to the preprocessed GMV images, with the cognitive reappraisal scores as a covariate of interest, and controlling for the age, sex, handedness, duration of education, and TIV covariates. To correct for multiple comparisons and test our hypothesis, we applied small volume correction (SVC), with a corrected *p*-value cutoff, using a binary mask image for the reward system. The binary mask was created by combining the VS and MPFC areas defined by a previous meta-analysis concerned with the valuation system in the human brain (Fig. 1C). Specifically, these regions were defined in accordance with the primary meta-analytic map reported by Bartra et al.^30^. This map represents a synthesis of fMRI literature on reward and decision-making, identifying neural loci where BOLD signal consistently covaries positively with subjective value (see Fig. 3D in Bartra et al., 2013; https://www.kablelab.com/resources.html).

### Mediation analysis

Based on the relationship found between cognitive reappraisal scores (with X as an independent variable) and the GMV of the left VS (with M as a mediator variable) (see “Results” below) and the relationship between cognitive reappraisal scores (X) and DD (with Y as a dependent variable), we performed a mediation analysis (Fig. 1A). This aimed to test whether the direct effect of cognitive reappraisal scores (X) on DD (Y) could be explained by the indirect influence of the GMV of the left VS (M). This analysis was performed using the Mediation Toolbox (https://github.com/canlab/MediationToolbox), which estimates direct, indirect, and total effects in mediation models. The toolbox employs bootstrapping to assess statistical significance of the indirect effect (a*b path), a recommended approach for mediation analyses. We used 10,000 bootstrap iterations to determine the significance of each path (*p* < 0.05)^45–47^. The participant variables age, sex, handedness, duration of education, and TIV were included in the model.

## Results

### Outcomes of behavioral and self-report measures

Our findings are based on individual *k*-scores derived from the DD task, where higher *k*-scores indicate a greater tendency to devalue future rewards, reflecting increased impulsive choice^2^. The mean (± standard deviation [SD]) *k*-score was 0.017 ± 0.023, and the mean log_10_*k* was − 2.002 ± 0.456. The mean (± SD) cognitive reappraisal and expressive suppression scores from ERQ were 27.632 ± 6.676 and 14.077 ± 4.624, respectively. Prior to our main analysis, we assessed the internal consistency of the questionnaire’s subscales within our sample. The Cronbach’s alpha values for both cognitive reappraisal (α = 0.843) and expressive suppression (α = 0.718) indicated high reliability.

### Relationship between emotional self-regulation strategies and delay discounting

**Fig. 2:**
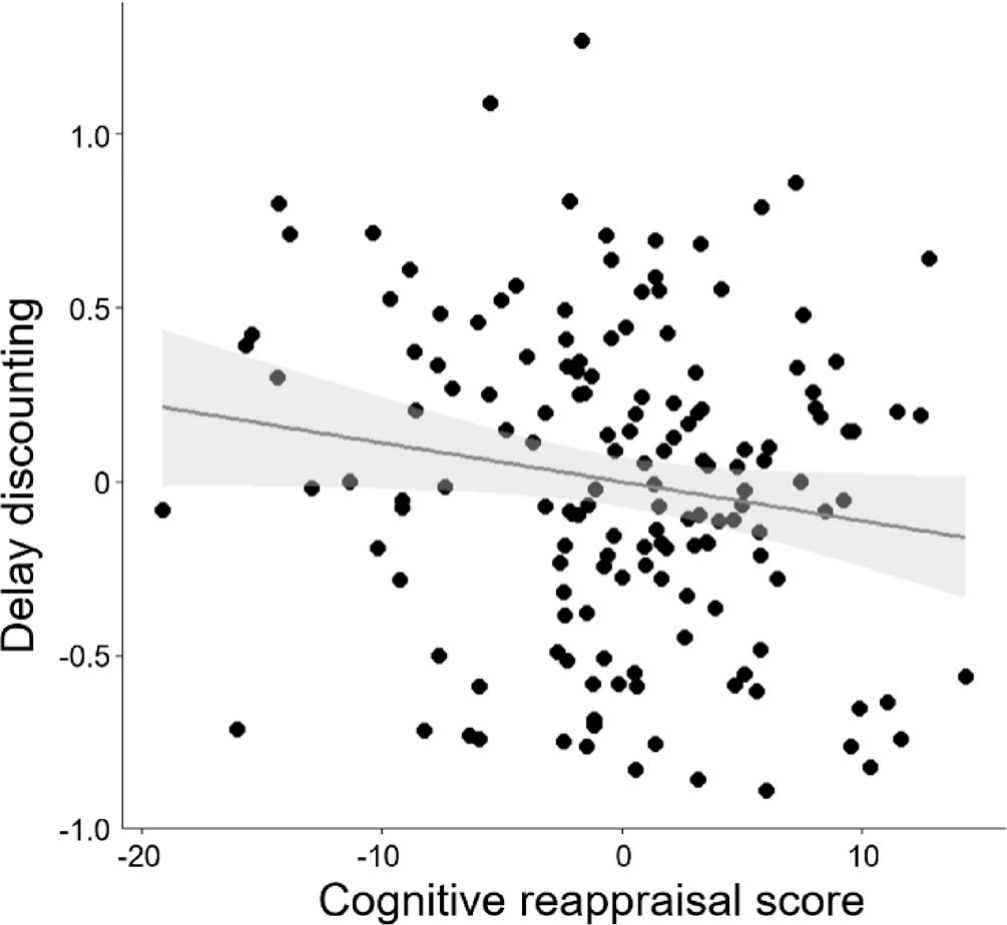
Association between cognitive reappraisal scores and delay discounting. Scatterplot of a correlation analysis between scores for cognitive reappraisal on the Emotion Regulation Questionnaire and delay discounting scores, after regressing out age, sex, IQ, and duration of education.

We found a significantly negative association between cognitive reappraisal scores and DD (*r* = − 0.171, *p* = 0.035) (Fig. 2), but no significant relationship between expressive suppression scores and DD (*r* = − 0.083, *p* = 0.314).

### Neuroanatomical correlates of cognitive reappraisal

**Fig. 3:**
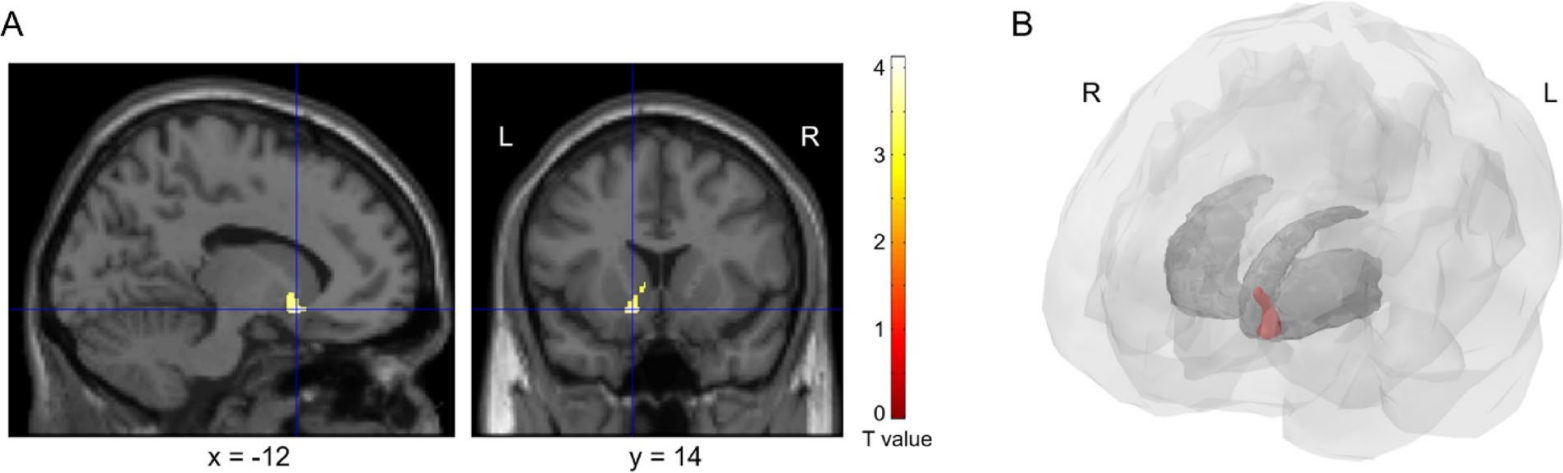
Brain areas associated with cognitive reappraisal. (**A**) When we applied small volume correction with a binary mask for the brain’s reward system to correct for multiple comparisons, we found the gray matter volume of the left ventral striatum to be negatively associated with cognitive reappraisal scores; (**B**) 3D rendering of a cluster associated with cognitive reappraisal scores (red).

When applying small-volume correction (SVC) with a binary mask to the reward system structures (VS and MPFC) for multiple comparison correction, a significant negative association was observed between cognitive reappraisal scores and the GMV of the left VS (Montreal Neurological Institute x, y, z coordinates = − 12, 14, − 10; *p* = 0.035; peak-level family-wise error (FWE)-corrected T = 3.94, 120 voxels) (Fig. 3A,B). However, when we corrected for multiple comparisons at the whole brain voxel level, no regions were significantly associated with cognitive reappraisal scores.

### Neuroanatomical mediators of the relationship between cognitive reappraisal and delay discounting

**Fig. 4:**
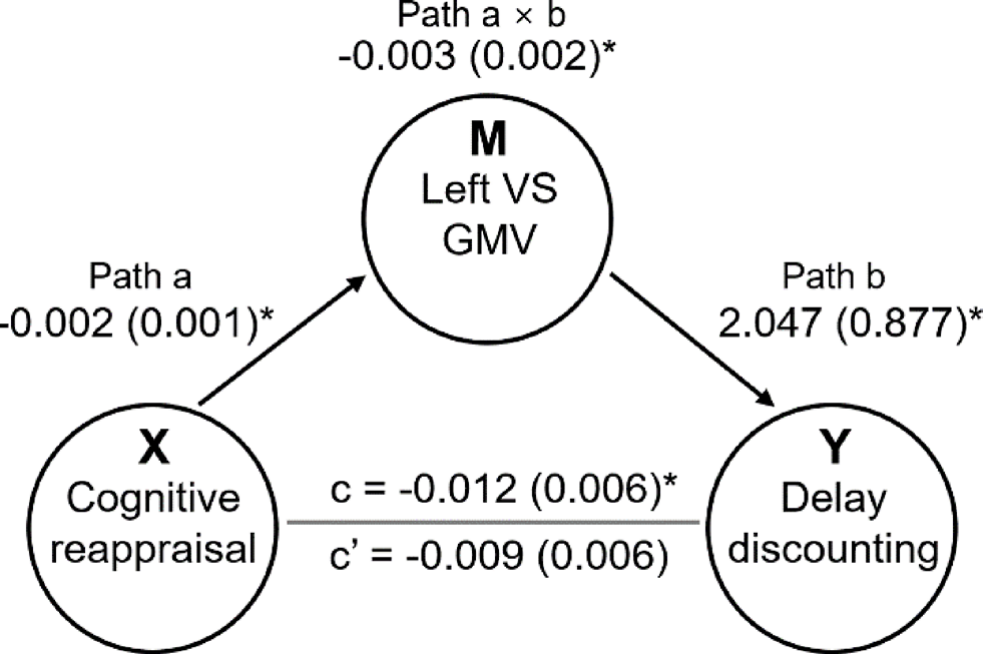
Negative mediation effect of the gray matter volume of the left ventral striatum on the relationship between cognitive reappraisal and delay discounting (path a × path b: coefficient = − 0.003, standard error [SE] = 0.002, *p* = 0.041). This was evidenced by cognitive reappraisal score-associated reductions in the GMV of the left VS (negative path a: coefficient = − 0.002, SE = 0.001, *p* = 0.012) and a correlation between the gray matter volume of the left ventral striatum and DD rates (positive path b: coefficient = 2.047, SE = 0.877, *p* = 0.019). GMV, gray matter volume; VS, ventral striatum.

A significant mediation effect was found between cognitive reappraisal, DD, and the GMV of the left VS) (Fig. 3). Cognitive reappraisal was negatively correlated with the GMV of the left VS (Fig. 4, Path a: coefficient = − 0.002, standard error [SE] = 0.001, *p* = 0.012). The GMV of the left VS was positively associated with DD (Fig. 4, Path b: coefficient = 2.047, SE = 0.877, *p* = 0.019). The direct effect of cognitive reappraisal on DD after controlling for left VS GMV was not significant (Fig. 4, Path c′: coefficient = − 0.009, SE = 0.006, *p* = 0.161). The total effect of cognitive reappraisal on DD (Path c) was significant (coefficient = − 0.012, SE = 0.006, *p* = 0.042). Finally, the GMV of the left VS showed a negative full mediation effect (Fig. 4, Path a × b: coefficient = − 0.003, SE = 0.002, *p* = 0.041), evidenced by the cognitive reappraisal score-associated reductions in the GMV of the left VS (Fig. 4, Negative Path a) and the positive relationship between the left VS GMV and DD rates (Fig. 4, Positive Path b).

## Discussion

This study is the first to investigate the emotional self-regulation strategies associated with DD, a measure of temporal impulsivity. Our analysis revealed a negative correlation between emotional self-regulation using cognitive reappraisal and DD. We also aimed to identify the neurological mediators of DD and emotional self-regulation. To achieve this, we examined the mediating effect of gray matter structures in the brain’s reward system on the association between cognitive reappraisal and DD. We found that higher cognitive reappraisal scores were negatively associated with the GMV of the left VS. Furthermore, higher GMV in the left VS was, in turn, positively associated with greater DD (i.e., increased impulsive choice). Our mediation analysis showed that this pattern of associations was significant, with the left VS GMV mediating the relationship between cognitive reappraisal and DD. Specifically, greater use of cognitive reappraisal was linked to smaller left VS GMV, and this smaller volume was associated with reduced DD. There was also a negative association between cognitive reappraisal and left VS GMV and a positive association between left VS GMV and DD. It is important to note, however, that while the pattern of results is statistically consistent with full mediation, the cross-sectional nature of our data precludes causal inference. These novel findings demonstrate that this structural component of the brain’s reward system is linked to cognitive reappraisal and impulsive decision-making.

Emotional self-regulation is crucial for controlling impulsive behavior, as it enables individuals to manage emotional responses, particularly in situations that might trigger impulsivity^48^. By focusing on long-term goals, effective emotional self-regulation helps individuals resist immediate temptations or urges that could lead to negative outcomes^49^. Our finding showing a negative association between emotional self-regulation and DD verifies the role of emotional self-regulation in the control of temporal impulsivity.

Our neuroimaging results show that the control of impulses through cognitive reappraisal is mediated by the left VS. Previous neuroimaging studies have identified other brain areas, such as the DLPFC, that are essential for top-down control over emotions and the inhibition of impulsive responses^50,51^. A recent meta-analysis demonstrated that noninvasive stimulation of the DLPFC influences decision-making, with excitation of the left DLPFC enhancing self-control in intertemporal and risk-related decisions^52^. These findings highlight the importance of specific brain regions in emotional self-regulation and suggest that enhancing the synaptic processes within them could be a valuable strategy for reducing impulsivity.

The VS is a key component of the brain’s reward system. It is crucial for processing reward- and motivation-related emotions^29^. Neuroimaging studies have consistently shown that the VS is activated during tasks involving emotional processing and reward anticipation^53,54^. Increased activation of this region has been found to enhance positive emotional experiences and increase motivation^55,56^. Dysregulation of VS processing can lead to difficulties managing emotions and increased impulsivity^57,58^. Effective emotional self-regulation depends on interactions between the VS and certain areas of the prefrontal cortex. Disruption of these interactions is associated with mood disorders and impaired emotional control^59,60^.

We initially hypothesized that both cognitive reappraisal and expressive suppression would be associated with DD. While we found a significant link between cognitive reappraisal and DD, no such association was observed with expressive suppression. Expressive suppression involves controlling the outward display of emotions. As such, it may not engage the reward system. This strategy inhibits emotional expression without altering the underlying emotional experience or evaluating possible rewards^61^. It has been shown to involve prefrontal structures such as DLFPC and the inferior frontal cortex, which are involved in self-control and behavioral inhibition, rather than the reward system, which is concerned with processing and anticipating positive outcomes^35,62^. Consequently, our results show a significant association between the reward system and cognitive reappraisal, but not with expressive suppression.

Our study revealed significant mediation effects in the relationships between cognitive reappraisal, DD, and GMV in the left VS (Fig. 4). While the link between the brain’s reward system (including the VS) and DD has been identified in previous studies^2,30^, the role of cognitive reappraisal in impulse control and its mediation by this brain region has not been thoroughly explored to date. Cognitive reappraisal is an effective emotional self-regulation strategy in which the individual reinterprets situations in ways that reduce their emotional impact, thereby decreasing impulsivity^61^. By modulating emotional reactions, cognitive reappraisal helps to maintain focus on long-term goals and increases resistance to immediate desires or urges. This is crucial to deliberate, well-considered decision-making. As mentioned earlier, VS plays a critical role in cognitive reappraisal and both are linked to impulse control. This study is the first to highlight the mediating role of the brain’s reward system, specifically the VS, in the relationship between cognitive reappraisal and impulsive behavior.

Interpreting the meaning of regional GMV differences, such as the observed negative association between cognitive reappraisal and left VS GMV, requires consideration of several factors, especially given the young adult cohort (mean age 22 years) who are still within a window of neurodevelopmental maturation. One possibility is that lower VS GMV in individuals with higher cognitive reappraisal reflects more advanced synaptic pruning or greater neural efficiency within this region^63,64^. Such processes are characteristic of brain maturation and can lead to more specialized and efficient neural circuits^65–67^. For example, Shaw and colleagues found that cognitive ability is closely linked to the developmental trajectory of GMV. Individuals with higher intelligence exhibit an initial increase in gray matter volume during early adolescence, followed by pronounced thinning in later adolescence—a pattern thought to reflect more efficient neural maturation and one that’s similar to our findings^68^. Similarly, Selemon’s review highlights that GMV reductions, largely driven by synaptic pruning during late adolescence and early adulthood, are associated with enhanced executive functions, including improved self-control^69^. These structural characteristics may underpin more adaptive functional responses within the VS during emotional regulation and reward processing, contributing to reduced impulsivity^70^. While these interpretations are speculative, they align with the understanding that brain development involves not just growth but also refinement of neural architecture^71^. Future longitudinal research tracking GMV changes alongside ER strategies and DD could elucidate these structure-function relationships and their developmental trajectories. Moreover, brain structure and function are bidirectionally related; persistent functional activity patterns associated with habitual cognitive reappraisal could plausibly induce long-term plastic changes in VS GMV, or conversely, pre-existing structural variations might bias individuals towards certain regulatory strategies^72^.

Our study had several notable strengths. First, it is a pioneering investigation into the relationship between impulsive behavior (as measured by DD rates) and emotional self-regulation by cognitive reappraisal and expressive suppression. It provides valuable insights into how these strategies relate to impulsive control and are mediated by neurological mechanisms. Second, the validity and reliability of our results are supported by previous findings showing an association between impulsive behavior and the VS^73,74^. The present study builds on our existing understanding of the role of the VS in cognitive reappraisal and impulsive decision-making. Our use of a large sample of 155 participants further bolstered the robustness and reliability of our findings.

However, there were also some limitations that should be addressed in future research. While we identified a significant mediation pathway, the cross-sectional nature of the study prevents us from determining the causal direction of the observed relationships. It is unclear whether habitual use of cognitive reappraisal induces plastic changes in the ventral striatum over time, or if pre-existing structural variations in the VS predispose individuals toward certain emotional regulation strategies. Second, our findings may not generalize beyond the specific measures used; DD specifically captures temporal impulsivity, which is distinct from other forms of impulsivity, and our reliance on self-report questionnaires for emotion regulation calls for further validation. Third, the sample consisted primarily of young adults, which limits the generalizability of the results to a broader age range and other demographics. Lastly, our neuroimaging findings regarding the association between cognitive reappraisal scores and gray matter volume were observed using SVC within our priori regions of interest (VS and MPFC). The use of SVC was theoretically driven by strong hypotheses about the involvement of these specific regions in reward processing and emotion regulation. This approach enhances statistical power for detecting effects in these targeted areas but means that we cannot ascertain effects outside of this network. Future research should be designed to directly address these limitations. A longitudinal study tracking a large, demographically diverse sample over time would be invaluable. Such a design could help disentangle the causal relationships between the habitual use of emotion regulation strategies, changes in brain structure, and their impact on impulsive decision-making throughout different life stages. Furthermore, to ensure findings are not confined to specific questionnaires, subsequent studies should incorporate a wider array of measures, including behavioral tasks, to provide a more comprehensive assessment of emotion regulation. Finally, future studies with larger samples would have the statistical power required to move beyond the SVC approach and perform comprehensive, whole-brain analyses to create a more complete map of the neural networks involved. Such research would build upon our findings to further refine the biomarkers that could predict individual differences in impulse control and guide the development of targeted interventions.

To our knowledge, this is the first novel study to report an association between cognitive reappraisal and impulsive behavior, as measured by DD, and the mediating effect of the brain’s reward system, particularly the VS, on this association. Our results align with previous findings regarding the relationship between these brain structures and impulsivity and highlight the crucial role the VS plays in linking cognitive reappraisal with impulse behavior. Based on these findings, we anticipate that manipulation of the structure of the VS, using methods such as brain stimulation or lesions, could be used in the future to influence emotional self-regulation strategies and impulse control. Further refined and validated, the biomarkers identified in this study could eventually be used in the prediction of individual differences in impulse control and the development of strategies for its enhancement.

## Data Availability

The datasets generated and analyzed for the present study are available from the corresponding author on reasonable request. To promote open science and increase the work’s visibility, the group-level maps are deposited on https://neurovault.org/collections/KAJOYOCF/.
